# Identification of Intestinal Microbial Community in Gallstone Patients with Metagenomic Next-Generation Sequencing

**DOI:** 10.3390/diagnostics13162712

**Published:** 2023-08-21

**Authors:** Li Ding, Su Wang, Wenrong Jiang, Yingxin Miao, Wenjian Liu, Feng Yang, Jinghao Zhang, Wenjing Chi, Tao Liu, Yue Liu, Shiwen Wang, Yanmei Zhang, Hu Zhao

**Affiliations:** 1 Department of Laboratory Medicine, Huadong Hospital Affiliated to Fudan University, Shanghai 200040, China; 2 Department of Laboratory Medicine, Research Center on Aging and Medicine, Fudan University, Shanghai 200040, China; 3 Department of Laboratory Medicine, Huadong Hospital, Shanghai 200040, China

**Keywords:** intestinal microbial community, gallstone disease (GD), species composition, microbial function

## Abstract

Gallstone disease (GD) is one of the most common gastrointestinal diseases worldwide. Nowadays, intestinal microbiota are thought to play important roles in the formation of gallstones. In our study, human fecal samples were extracted for metagenomic next-generation sequencing (mNGS) on the Illumina HiSeq platform, followed by bioinformatics analyses. Our results showed that there was a particular intestinal micro-ecosystem in GD patients. In contrast to healthy people, the sequences of *Bacteroidetes*, *Bacteroides* and *Thetaiotaomicron* were obviously more abundant in GD patients at phylum, genus and species levels, respectively. On the other hand, the glycan metabolism and drug resistance, especially for the β-lactams, were the most profound functions of gut microbes in GD patients compared to those in normal subjects. Furthermore, a correlation analysis drew out that there existed a significant relationship between the serum levels of biochemical indicators and abundances of intestinal microbes in GD patients. Our results illuminate both the composition and functions of intestinal microbiota in GD patients. All in all, our study can broaden the insight into the potential mechanism of how gut microbes affect the progression of gallstones to some extent, which may provide potential targets for the prevention, diagnosis or treatment of GD.

## 1. Introduction

Gallstone disease (GD), also known as cholelithiasis, is a common disease which can stimulate the gallbladder mucosa and result in acute/chronic cholecystitis or even gallbladder carcinoma [1]. The cholesterol gallstone is the most familiar type of GD in cholecystectomy [2]. The prevalence of GD is extremely high in Western countries with the rate of about 10~20% [3]. Nowadays, GD has become more and more prevalent in China, ranging from 10 to 15% [4]. The majority of the population with the disease are women and elderly people. Generally, the morbidity of GD can be impacted by a great deal of factors, including heredity, lifestyle, dyslipidemia and especially a high-cholesterol diet [5,6]. The abnormal metabolism or supersaturated secretion of cholesterol and bile acids is commonly believed to induce the formation of gallstones [7,8].

It is universally acknowledged that intestinal microbial communities participate in regulating the endocrine and biological metabolism in human bodies [9,10,11], which are intimately associated with various diseases, such as adiposity, diabetes, inflammation, depression or even some kinds of tumors [12,13,14,15]. In recent years, several researchers have suggested that intestinal microbiota may play a vitally important role in gallstone pathogenesis [16,17]. Wang et al. supposed that a lithogenic diet could lead to dramatic alteration in the abundance and composition of gut microbiota, which might contribute to the metabolic disorders of cholesterol and bile acid [17]. Wu et al. found an overgrowth of the bacterial phylum *Proteobacteria* within the gut of GD patients, while three gut bacterial genera, including *Faecalibacterium*, *Lachnospira* and *Roseburia*, significantly decreased [18]. Interestingly, Keren et al. pointed out that the intestinal genus *Roseburia* and the species *Bacteroides* were reduced, but the family *Ruminococcaceae* and the genus *Oscillospira* increased in GD patients [19]. However, the pathogenesis of GD affected by intestinal microbiota still remained unclear up until now. The most common hypothesis could be concluded that bile acids’ metabolism is mediated by intestinal bacteria via the activation of bile salt hydrolases (BSH), existing in genera *Bifidobacterium*, *Lactobacillus*, *Clostridium*, etc. BSH might further dissociate both 7α-dehydroxylase and bile acids, thereby turning primary bile acids into secondary bile acids. The high level of secondary bile acids is considered to cause an increased secretion of biliary cholesterol and formation of gallstones [20,21,22]. On the whole, the available studies usually focused on the description of species in cholelithic gut microbiota with 16S rRNA sequencing. Very few of them laid emphasis on the detailed function of those differential microbes. In view of the intestinal microbial community being a complex and crucial ecosystem, more and more research should be conducted to reveal the intrinsic effect of gut microbiota on the occurrence and development of GD.

In our study, metagenomic next-generation sequencing (mNGS) was performed on the Illumina HiSeq platform so as to undertake a relatively comprehensive analysis of the relationship between intestinal microbiota and GD. In summary, we attempted to draw a clear illustration of four important and key issues: (1) The characteristics of the intestinal microbial community in GD patients compared with those in healthy individuals. (2) The functions of differential gut microbiota in GD patients. (3) The relationship between intestinal microbiota and traditional biochemical markers in patients with cholelithiasis. (4) The potential mechanism of how the intestinal microbial community affects the formation of gallstones.

## 2. Materials and Methods

### 2.1. Patient Cohorts

GD patients and healthy individuals in our study were all recruited from Huadong Hospital affiliated with Fudan University. The criteria used for the selection of patients were as follows: (1) The diagnostic criteria were according to the European Association for the Study of the Liver (EASL) guidelines. (2) None of the patients indicated they had suffered gastrointestinal diseases except GD. (3) All the patients were excluded from chronic diseases, such as cirrhosis, diabetes, cardiovascular disease, etc. (4) None of the patients had taken antibiotics or probiotics within the previous 3 months prior to this study. (5) None of the patients underwent surgery prior to this study. 

In all selected cases, the characteristics of healthy individuals were as follows: (1) None of the healthy individuals had suffered any diseases of the gastrointestinal tract or other chronic diseases. (2) None of them had been subjected to surgical procedures for several years prior to this study. (3) None of them had taken antibiotics or probiotics within the previous 3 months prior to this study. 

Our study was approved by the committee for ethical review of research involving human subjects (Ethical Project No. 2018k045), Huadong Hospital affiliated with Fudan University, Shanghai, China. All participants signed informed consent forms.

### 2.2. Fecal Samples’ Collection

Fresh fecal samples were obtained from the GD patients or healthy individuals. The collection procedures were followed by our previously published methods [23,24]. All the fecal samples were placed in cryovials without a preservative, then immediately snap-frozen in liquid nitrogen and stored at −80 °C. Afterwards, the samples were kept on dry ice for the subsequent sequencing analysis. All samples were stored in their original tubes at −80 °C until further processing.

### 2.3. DNA Extraction and Sequencing

A DNeasy PowerSoil Kit (QIAGEN, Inc., Dusseldorf, Germany) was used for extracting the total microbial genomic DNA from fecal samples. The extraction procedure was conducted under the guidance of the manufacturer’s instructions. Then, the quality and quantity of extracted DNA were estimated with agarose gel electrophoresis and a NanoDrop spectrophotometer (ND-1000, Thermo Fisher Scientific, Waltham, MA, USA), respectively. After that, an Illumina TruSeq Nano DNA LT Library Preparation Kit (Illumina, San Diego, CA, USA) was used to set up metagenome shotgun sequencing libraries with insert sizes of 400 bp using extracted microbial DNA. Finally, the sequencing processes of constructed libraries were performed on the Illumina HiSeq X-ten platform (Illumina, USA) with a PE150 strategy at Personal Biotechnology Co., Ltd. (Shanghai, China).

### 2.4. Sequence Analysis

Raw sequencing reads were processed to obtain quality-filtered reads for the further analysis. First of all, Cutadapt (v1.2.1) was used to eliminate sequencing adapters from sequencing reads [25]. Then, low-quality reads were cleaned up with a sliding-window algorithm. Thirdly, qualified reads were aligned to the host genome with a Burrows Wheeler Alignment (BWA) Tool (http://bio-bwa.sourceforge.net/) (accessed on 18 April 2022) to clear host contamination [26]. The reads were further applied to construct the metagenome for each sample when they were de novo assembled with an iterative De Bruijn graph assembler for sequencing data with a highly uneven depth (IDBA-UD) [27]. Finally, the coding regions (CDS) of metagenomic scaffolds (>300 bp) were predicted with MetaGeneMark (http://exon.gatech.edu/GeneMark/metagenome) (accessed on 18 April 2022) [28], followed by CDS sequence clustering so as to obtain a non-redundant gene catalog [29]. 

The sequence data analyses were mainly performed using R packages (v3.2.0). Operational Taxonomic Units (OTU)-level alpha diversity indices, such as the Chao1 richness estimator, abundance-based coverage estimator metric (ACE), Shannon diversity index and Simpson index, were calculated using the OTU table in Quantitative Insights Into Microbial Ecology (QIIME). Meanwhile, a beta diversity analysis was performed to investigate the compositional and functional variation of microbial communities of all samples using Bray–Curtis distance metrics and visualized via a principal coordinate analysis (PCoA) [30], nonmetric multidimensional scaling (NMDS) and the unweighted pair-group method with arithmetic means (UPGMA) hierarchical clustering [31]. Additionally, the functional profiles of the non-redundant genes were obtained by annotating against the Gene Ontology (GO), Kyoto Encyclopedia of Genes and Genomes (KEGG), Evolutionary genealogy of genes: Non-supervised Orthologous Groups (EggNOG) and Carbohydrate-Active enzymes (CAZy) databases, respectively, by using the double index alignment of next-generation sequencing data (DIAMOND) alignment algorithm [32]. Based on the taxonomic and functional profiles of non-redundant genes, linear discriminant analysis effect size (LEfSe) was used to detect differentially abundant taxa and functions across groups using the default parameters [33]. Moreover, a random forest analysis was applied for discriminating different samples using the R package “random Forest” with 1000 trees and all default settings [34,35]. The generalization error was estimated using 10-fold cross-validation. The expected “baseline” error was also included, which was obtained with a classifier that simply predicted the most common category label.

### 2.5. Data Access

All raw sequences were deposited in the NCBI Sequence Read Archive (SRA) under the accession number PRJNA999028.

### 2.6. Statistical Analysis

Statistical analyses were performed with R packages (v3.2.0) and SPSS version 20.0 (SPSS, Chicago, IL, USA). The comparisons of species and related functions between groups were displayed with the LEfSe method. Differences of clinical features between groups were analyzed with a one-way ANOVA or Chi-square test. Correlation analyses were conducted with a Pearson’s correlation test. The degree of correlation was evaluated with the Pearson correlation coefficient. In all cases, *p* < 0.05 was considered to be statistically significant.

## 3. Results

### 3.1. Analysis of Intestinal Microbial Community in GD Patients

A total of 62 fecal samples from 42 GD patients (16 males/26 females) and 20 healthy individuals (12 males/8 females) were included in our study. The values of serum biochemical markers and parameter distribution of all samples are shown in Table 1. The Scaffolds/Scaftigs of each sample were aligned using BLASTN with the sequences of Bacteria, Archaea, Fungi and Viruses in the NCBI-NT database (Nucleotide collection, ftp://ftp.ncbi.nih.gov/blast/db/) (accessed on 21 February 2023), followed by an analysis of species classification from phylum to species on the MEtaGenome Analyzer platform (http://ab.inf.uni-tuebingen.de/software/megan5) (accessed on 21 February 2023) according to the lowest common ancestor (LCA) algorithm [36,37].

**Figure 1:**
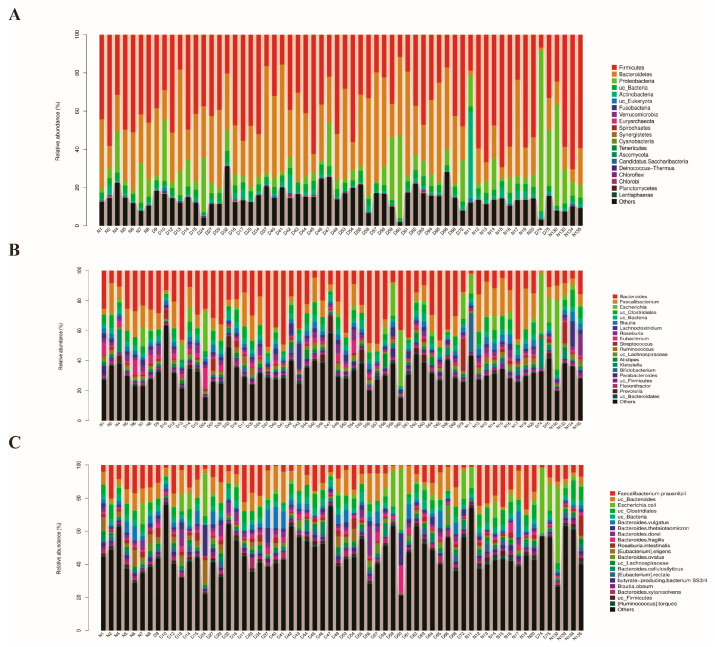
The composition of intestinal microbiota in GD patients and healthy individuals. (**A**) The composition of intestinal microbiota at the phylum level. (**B**) The composition of intestinal microbiota at the genus level. (**C**) The composition of intestinal microbiota at the species level. Note: N: healthy individuals, D: GD patients.

In general, the intestinal microbial composition of GD patients was described with taxonomic profiling. At the phylum level, we noticed *Firmicutes* (36.13%), *Bacteroidetes* (31.85%), *Proteobacteria* (10.71%) and *Actinobacteria* (1.66%) accounted for the majority of the sequences (Figure 1A). When it comes to the genus level, *Bacteroides* (26.17%), *Faecalibacterium* (6.99%), *Escherichia* (5.12%), *Blautia* (2.30%) and *Lachnoclostridium* (2.44%) were found to be the main gut genera for GD patients (Figure 1B). Furthermore, *Faecalibacterium prausnitzii* (6.9%), *Escherichia coli* (5.1%), *Bacteroides vulgatus* (3.5%), *Bacteroides thetaiotaomicron* (2.7%), *Bacteroides dorei* (2.5%), *Bacteroides fragilis* (2.3%), *Roseburia intestinalis* (1.5%) and *Bacteroides cellulosilyticus* (1.4%) were the dominant gut microbes (>1% of all sequences) at the species level (Figure 1C). Additionally, no matter in the GD patients or in the healthy individuals, there are no newly identified microbes.

**Figure 2:**
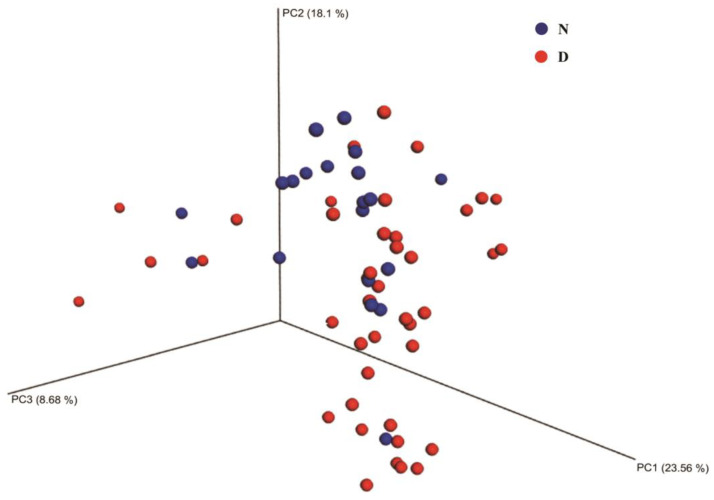
Bray–Curtis-distance-based PCoA of species composition dissimilarity between GD patients and healthy people. The percentages in the axes represent the proportion of differences in the original data, which the corresponding principal coordinates can explain. Each point represents a sample and points of different colors belong to different groups. Note: N: healthy individuals, D: GD patients.

Moreover, the beta diversity of species composition was estimated with Bray–Curtis-distance-based PCoA. The result revealed the fecal samples from GD patients were grouped together showing obviously less similarities to each other than to those samples from healthy individuals (Figure 2).

### 3.2. The Intestinal Microbiota in GD Patients Were Extraordinarily Different from Those in Healthy Individuals

**Figure 3:**
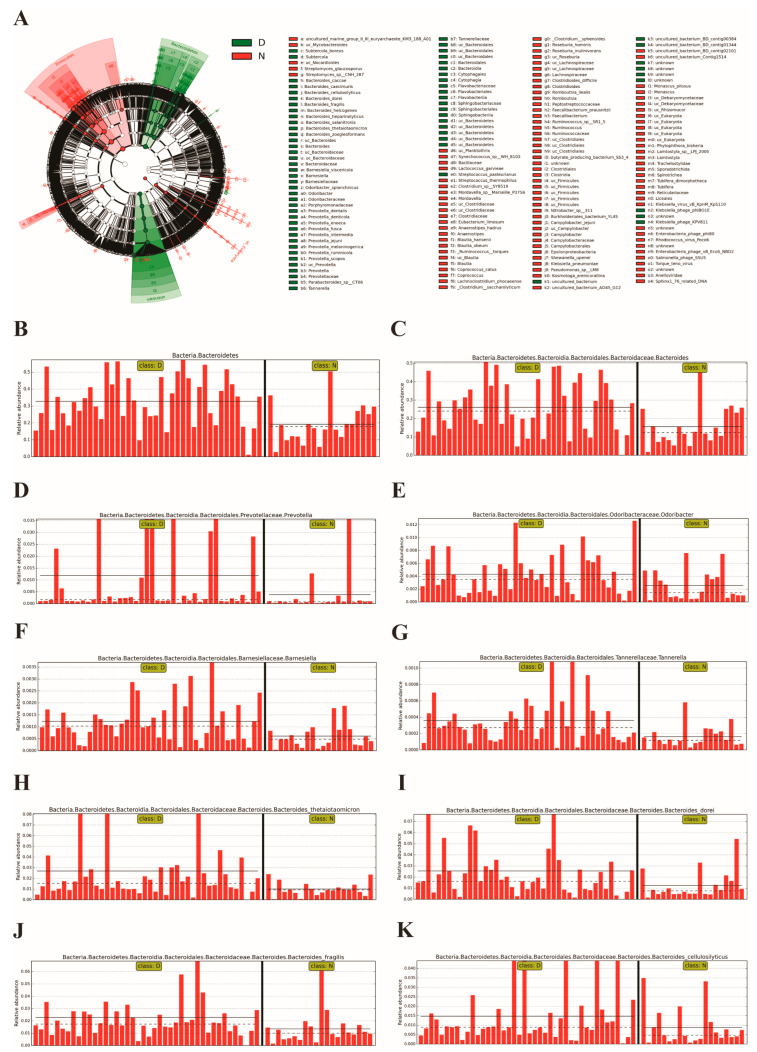
Analysis of the specific intestinal microbes in GD patients compared with those in healthy individuals with the LEfSe method. (**A**) The taxonomic rank shows the subordination of the species in turn from the inner circle to the outer circle. The node size corresponds to the average relative abundances of species. The node color indicates the species with significant dissimilarities between groups. The names of different species are identified using letters. (**B**–**K**) Intestinal microbes with the most significant difference in the patient group compared to those in healthy people. Note: N: healthy individuals, D: GD patients.

To elucidate the specific gut microbiota of GD patients, the LEfSe method was conducted on the Galaxy online analysis platform (http://huttenhower.sph.harvard.edu/galaxy/) (accessed on 21 February 2023) according to the species composition spectra. The results showed that there were several kinds of intestinal microbes with a significant difference in the patient group compared to those in healthy individuals at the phylum, genus and species levels, respectively. To be specific, the member of phylum *Bacteroidetes* (logarithm value: 5.515, *p* = 0.001) was the only differential species in GD patients. At the genus level, *Bacteroides* (5.418, *p* = 0.004), *Prevotella* (4.075, *p* < 0.001), *Odoribacter* (3.636, *p* = 0.027), *Barnesiella* (3.091, *p* = 0.003), *Tannerella* (2.557, *p* < 0.001), etc., were more frequently detected in GD patients. In addition, the main bacterial species were represented by *Thetaiotaomicron* (4.430, *p* < 0.01), *Dorei* (4.407, *p* < 0.05), *Fragilis* (4.359, *p* < 0.01), *Cellulosilyticus* (4.167, *p* < 0.05), *Salanitronis* (3.761, *p* < 0.01), etc., in the fecal microecosystem of the patients (Figure 3).

### 3.3. The Functions of Intestinal Microbiota in GD Patients Varied from Those in Healthy Individuals

The LEfSe method was used to further explore the functions of differential gut microbes in GD patients according to the abundance spectra of basic functional groups of all samples annotated in the KEGG database [38]. The results revealed that the gut microbial function could be divided into several sections, including metabolism, human diseases, cellular processes and organismal systems.

**Figure 4:**
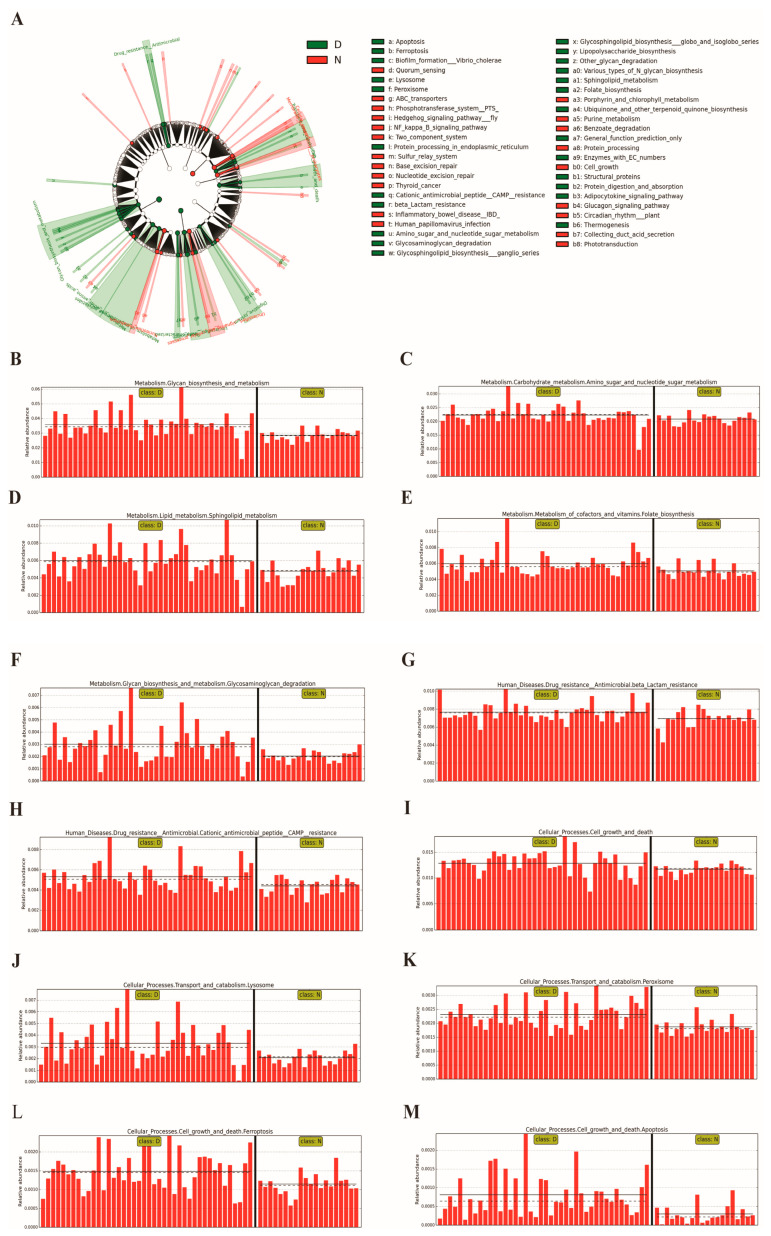
Analysis of the key function of intestinal microbiota in GD patients compared to that in healthy people with the LEfSe method. (**A**) The taxonomic rank shows the subordination of the functional taxa in turn from the inner circle to the outer circle. The node size corresponds to the average relative abundance of functional taxa. The node color indicates the functional taxa with significant dissimilarities between groups. The names of different functional taxa are identified using letters. (**B**–**M**) Microbial functions with the most significant differences in the patient group compared to those in healthy individuals. Note: N: healthy individuals, D: GD patients.

In the GD group, within the metabolism section, glycan biosynthesis (logarithm value: 4.554, *p* < 0.001), amino sugar and nucleotide sugar metabolism (4.348, *p* = 0.023), sphingolipid metabolism (3.777, *p* = 0.004), folate biosynthesis (3.775, *p* = 0.008) and glycosaminoglycan degradation (3.479, *p* = 0.004), etc., showed a remarkably higher proportion compared with those in healthy individuals. When it comes to the human diseases section, only the antimicrobial resistance class exhibited an obviously higher representation in GD patients. Moreover, the β-lactam resistance (3.884, *p* < 0.014) and cationic antimicrobial peptide (CAMP) resistance (3.727, *p* = 0.007) were the two sub-classes with significant differences. When it comes to the cellular processes section, the cholelithic gut microbiota were involved in cell growth and death (4.109, *p* = 0.008), lysosome transport and catabolism (3.520, *p* < 0.002), peroxisome transport and catabolism (3.366, *p* < 0.001), ferroptosis (3.170, *p* = 0.008) and apoptosis (2.909, *p* < 0.001). Finally, within the organismal systems section, the intestinal microbial community in GD patients was found to participate in regulating the environmental adaptation, endocrine system and digestive system function, especially the adipocytokine signaling pathway (3.109, *p* = 0.030), thermogenesis (3.097, *p* = 0.024) and protein digestion and absorption (2.777, *p* = 0.002) (Figure 4).

### 3.4. The Species and Functions with the Highest Discriminatory Power of Intestinal Microbiota in GD Patients

The random forest analysis was performed to figure out the species and functions with the highest discriminatory power of intestinal microbiota in GD patients [39]. Our results showed that *Sphingobacterium* sp. G1-14, uncultured *Agaricomycetes*, uncultured *Agaricales*, *Exiguobacterium* sp. 11–28, *Gymnopus* sp. VC-2017f, *Eubacterium ramulus*, *Faecalibacterium* sp., *Rhizomucor miehei*, *Acinetobacter nosocomialis* and *Enterobacter* sp. *Crenshaw* were the top 10 species in the patient group (Table 2). On the other hand, the secondary metabolites’ biosynthesis, defense mechanisms, transcription, amino acid transport and metabolism, inorganic ion transport and metabolism, intracellular trafficking, secretion and vesicular transport, coenzyme transport and metabolism, cell cycle control, cell division and chromosome partitioning, energy production and conversion, post-translational modification and protein turnover were the top 10 biological functions of the gut microbes (Table 3).

### 3.5. The Levels of Serum Biochemical Indicators Were Correlated with the Abundances of Intestinal Microbes in GD Patients

The serological detection is usually an adjunctive method for diagnosing cholelithiasis. In order to explore whether the serum biochemical indicators are related to the abundances of intestinal microbes in cholelithiasis subjects, the relevant information of such indicators including total bilirubin, direct bilirubin, total bile acid, alkaline phosphatase, γ-glutamyl transpeptidase, cystatin C, prealbumin, lactate dehydrogenase, glutamate dehydrogenase, etc., was collected. In addition, only the top 10 differential species screened out by LEfSe were subsumed for the analysis. A Spearman’s rank correlation analysis showed that there was a positive correlation between the abundance of *Thetaiotaomicron* and the concentration of serum prealbumin (r = 0.483, *p* = 0.027). However, the concentration of serum total bilirubin was negatively correlated with the abundance of both *Dorei* (r = −0.395, *p* = 0.017) and *Cellulosilyticus* (r = −0.416, *p* = 0.012), and the abundance of *Fragilis* was negatively correlated with the concentration of serum cystatin C (r = −0.402, *p* = 0.027) (Table 4).

## 4. Discussion

GD is recognized as a significant global health problem. At present, the prevalence of cholelithiasis keeps a constantly rising tendency, accompanied by the tremendous growing financial burden. It was reported that a great many intrinsic or extrinsic factors could contribute to GD [5,6]. The metabolic disturbances of cholesterol and bile acid are considered to be the key factors among them. However, the potential pathogenic mechanisms of gallstone formation still need to be illuminated.

To date, the microecosystem of the human intestinal tract has been widely studied. In recent years, some studies have explored the gut microbial community of GD patients with 16S rRNA amplicon sequencing [18,40]. However, the majority of them usually lay emphasis on the species composition or biological diversity of the microbiota. In our study, mNGS was used to describe the characteristics of cholelithic gut microbiota with GD patients. We not only focused on the composition and diversity of the microbes, but also explored their functions in the human intestinal ecosystem.

Additionally, we found that the intestinal tract of GD patients harbored a particular microbial community using bioinformatic analyses. In general, the intestinal microbiota were composed of four kinds of phyla including *Firmicutes*, *Bacteroidetes*, *Proteobacteria* and *Actinobacteria*, and one absolutely predominant genus *Bacteroides* and several species that shared analogous abundances like *Faecalibacterium prausnitzii*, *Escherichia coli*, *Bacteroides thetaiotaomicron*, etc. Such findings are similar to the studies of Keren et al. [19]. Interestingly, some researchers pointed out that the biliary microbiota in patients with gallbladder gallstones were represented by *Bacteroidetes*, *Firmicutes* and *Proteobacteria* at the phyla level and *Bacteroides* at the genera level, respectively, which indicated the biliary microbial distribution was almost in accordance with that in the intestine [41,42]. In view of a gut pathogen infection as one of the most significant factors to induce the occurrence and development of GD, we hypothesized that the microbes colonizing in gallbladders of GD patients might practically immigrate from the human intestinal tract. Obviously, there is a great deal of difference between the intestinal and biliary tract structures. Some microbes have to change their characteristics or metabolic activities so as to adapt to the new environment after the immigration. In this way, they might produce a few pathogenic or invasive metabolites, which can result in the disturbance of biliary functions.

Moreover, a random forest analysis was used to explore the intestinal species with the highest discriminatory power in GD patients. After sorting them according to the importance of the species, we found that *Sphingobacterium* sp. G1-14, *uncultured Agaricomycetes* and *uncultured Agaricales* were the three most vitally important microbes, which could be considered as the markers of the intestinal microbial community in GD patients. Additionally, the PCoA analysis distinguished the microbial similarity between two groups, showing a notably higher dispersion among the samples from GD patients. In particular, the microbial communities significantly differed from each other even among GD patients, which indicated that the composition of intestinal microbes in GD patients was quite various and complex. On the contrary, the microbes in the intestinal tract of healthy people were relatively stable and homogeneous.

Since the composition of intestinal microbiota was different between two groups, it was rather essential to figure out the exact microbes. Thus, the LEfSe method was applied for the further identification. In addition to the sequences matching the phylum *Bacteroidetes* and the genera *Bacteroides*, *Prevotella*, *Odoribacter*, etc., we observed that some particular species including *Bacteroides thetaiotaomicron*, *Bacteroides Fragilis*, *Bacteroides Cellulosilyticus*, etc., were remarkably more abundant in GD patients. Hence, we supposed that such species could be closely associated with the pathological conditions of cholelithiasis. For instance, *Bacteroides fragilis* belongs to bile-tolerant microbes as well as opportunistic pathogens. An opportunistic pathogen is an infectious pathogen that is normally commensal in the body but can colonize elsewhere and cause an infectious disease by taking advantage of the weakened immunity of the host or gut dysbacteriosis. *Bacteroides fragilis* can migrate from the gut to the biliary tract or gallbladder when the body suffers from impaired immunity or gut dysbacteriosis, which is caused by various internal and external factors. Thanks to its tolerance to bile, *Bacteroides fragilis* can stably inhabit in the biliary tract or gallbladder and may even induce the infection of the biliary system, promoting the formation of gallstones. 

Furthermore, we also figured out that the glycan metabolism and the β-lactam resistance were two predominant functions of the intestinal microbiota in GD patients analyzed with the LEfSe method. *Bacteroides thetaiotaomicron* is a gut commensal that mainly degrades carbohydrates and promotes the absorption of bile and cholesterol, contributing to gut physiology. The overgrowth of *Bacteroides thetaiotaomicron* can undoubtedly affect the balance of intestinal bile metabolism, resulting in bile acid dysmetabolism. Although the mechanism of bile acids affecting glycometabolism in the development of cholelithiasis still remains unclear, there was evidence that bile acids could inhibit the transcription of gluconeogenesis-related genes in a Farnesoid-X-receptor–Small-Heterodimer-Partner (FXR-SHP)-dependent manner [43]. In addition, researchers showed that bile acids could stimulate the expression of TGR5 as its ligand, and further lead to the activation of adenylate cyclase and protein kinase A, thus regulating the carbohydrate metabolism [44]. To sum up, *Bacteroides thetaiotaomicron* might participate in the formation of gallstones due to its role in bile acid dysmetabolism. On the other hand, it has come to light that the β-lactams are the commonly used antibiotics for the treatment of gallstone disease caused by pathogenic bacteria infection. Thus, we predict that one of the most important reasons for the difficulty in eradicating GD is probably the antibiotic resistance resulting from intestinal microbial disorders.

In addition, the correlation between the abundances of differential intestinal microbes and serum biochemical markers in GD patients was far more important for investigation. We observed that there was a positive correlation between the abundance of *Thetaiotaomicron* and the concentration of serum prealbumin. Most GD patients often suffer from malnutrition and some of them may have abnormal serum prealbumin levels. *Thetaiotaomicron* can decompose polysaccharides so as to provide energy for the biological metabolism [45]. Theoretically, both the *Thetaiotaomicron* abundance and serum prealbumin level can reflect whether the body is in a normal physiological state to a certain extent. Apart from that, we also found that the concentration of serum total bilirubin was negatively correlated with the abundances of *Dorei* and *Cellulosilyticus*, while the abundance of *Fragilis* was negatively correlated with the serum cystatin C level. However, more studies should be conducted to reveal the underlying mechanism regarding how these correlations were formed. We suppose that such microbes may participate in the oxidation and epimerization of bile acids, thus disrupting the enterohepatic circulation and leading to the formation of gallstones.

Finally, although we recruited normal individuals and patients according to the enrollment criteria, the limited number of healthy controls might be a limitation of our present research. It would be better to recruit more healthy people to enrich our findings. In our further study, we will attempt to expand the number of healthy subjects to validate our results, which will achieve a far more comprehensive assessment of the intestinal microbial community in GD progression. 

To sum up, our research elucidated the characteristics of the intestinal microbial community in GD patients and found the closely related species for them. Using a comparison with the healthy individuals, we discovered the differential intestinal microbes and the corresponding functions in cholelithiasis subjects. Meanwhile, we identified that the cholelithic intestinal microbiota were correlated with the traditional serum biochemical markers. All in all, our study opened up new strategies for drawing out the role of the intestinal microbial community in the progression of GD. Additionally, our results might reveal the underlying mechanisms of the occurrence or development of GD.

## 5. Conclusions

Our study revealed that the intestinal microbial community of GD patients was unique from that of healthy individuals. By means of the mNGS, we not only figured out the differential microbes of cholelithiasis but their functions as well. Moreover, the lithic species and corresponding functions with the highest discriminatory power were identified with a random forest analysis. Furthermore, the abundances of intestinal microbes were determined to be related to serum biochemical markers in GD patients. In conclusion, our study can broaden the insight into the potential mechanism of how gut microbes affect the progression of gallstones to some extent, which may provide potential targets for the prevention, diagnosis or treatment of GD.

## Figures and Tables

**Table 1:** Clinical features of the samples.

	GD Patients	Healthy People	*p*-Value
Age (years)	49.69 ± 7.37	46.10 ± 6.32	0.066
Gender			
Male	16	12	0.105
Female	26	8	
Glutamate Dehydrogenase (μmol/L)	3.39 ± 2.10	-	-
Total Protein/Albumin	1.62 ± 0.46	-	-
Total Protein (μmol/L)	71.89 ± 5.00	-	-
Albumin (μmol/L)	45.08 ± 3.52	-	-
Prealbumin (μmol/L)	233.37 ± 41.85	-	-
Alanine Aminotransferase (μmol/L)	25.16 ± 18.51	-	-
Aspartate Aminotransferase (μmol/L)	22.73 ± 18.64	-	-
Lactate Dehydrogenase (μmol/L)	168.81 ± 25.12	-	-
Total Bile Acid (μmol/L)	4.10 ± 3.10	-	-
γ-Glutamyl Transpeptidase (μmol/L)	45.69 ± 23.96	-	-
Direct Bilirubin (μmol/L)	4.49 ± 2.40	-	-
Total Bilirubin (μmol/L)	11.45 ± 5.01	-	-
Alpha-l-fucosidase (μmol/L)	19.85 ± 5.47	-	-
Cystatin C (μmol/L)	0.73 ± 0.18	-	-

**Table 2:** Species with the highest discriminatory power (top 10) of intestinal microbiota in GD patients.

Feature ID	Mean Decrease in Accuracy	Standard Deviation
k__*Bacteria*; p__*Bacteroidetes*; c__*Sphingobacteriia*; o__*Sphingobacteriales*; f__*Sphingobacteriaceae*; g__*Sphingobacterium*; s__*Sphingobacterium* sp. G1-14	0.001162928	0.000606256
k__*Eukaryota*; p__*Basidiomycota*; c__*Agaricomycetes*; o__uc_*Agaricomycetes*; f__*uc_Agaricomycetes*; g__uc_*Agaricomycetes*; s__uc_*Agaricomycetes*	0.001031263	0.000604338
k__*Eukaryota*; p__*Basidiomycota*; c__*Agaricomycetes*; o__*Agaricales*; f__uc_*Agaricales*; g__uc_*Agaricales*; s__uc_*Agaricales*	0.000936848	0.00046726
k__*Bacteria*; p__*Firmicutes*; c__*Bacilli*; o__*Bacillales*; f__unknown; g__*Exiguobacterium*; s__*Exiguobacterium* sp. 11–28	0.000832036	0.000560922
k__*Eukaryota*; p__*Basidiomycota*; c__*Agaricomycetes*; o__*Agaricales*; f__*Omphalotaceae*; g__*Gymnopus*; s__*Gymnopus* sp. VC-2017f	0.000810951	0.000455266
k__*Bacteria*; p__*Firmicutes*; c__*Clostridia*; o__*Clostridiales*; f__*Eubacteriaceae*; g__*Eubacterium*; s__*Eubacterium ramulus*	0.000759662	0.000347172
k__*Bacteria*; p__*Firmicutes*; c__*Clostridia*; o__*Clostridiales*; f__*Ruminococcaceae*; g__*Faecalibacterium*; s__*Faecalibacterium* sp.	0.000712143	0.000622133
k__*Eukaryota*; p__*Mucoromycota*; c__*Mucoromycetes*; o__*Mucorales*; f__*Lichtheimiaceae*; g__*Rhizomucor*; s__*Rhizomucor miehei*	0.000711614	0.000220115
k__*Bacteria*; p__*Proteobacteria*; c__*Gammaproteobacteria*; o__*Pseudomonadales*; f__*Moraxellaceae*; g__*Acinetobacter*; s__*Acinetobacter nosocomialis*	0.000686536	0.000425183
k__*Bacteria*; p__*Proteobacteria*; c__*Gammaproteobacteria*; o__*Enterobacterales*; f__*Enterobacteriaceae*; g__*Enterobacter*; s__*Enterobacter* sp. Crenshaw	0.000654157	0.000538885

**Table 3:** Microbial functions with the highest discriminatory power (top 10) of intestinal microbiota in GD patients.

Feature ID	Mean Decrease in Accuracy	Standard Deviation
S Function unknown; ENOG4107YKV	0.000523052	0.000482557
Q Secondary metabolites biosynthesis, transport and catabolism; ENOG4107VZP	0.000514268	0.000594528
S Function unknown; ENOG4106UH8	0.000445652	0.000245257
V Defense mechanisms; ENOG4107RKB	0.000408646	0.000340324
K Transcription; ENOG4105S4D	0.00038124	0.000329398
E Amino acid transport and metabolism; arCOG05229	0.000348469	0.000414534
S Function unknown; ENOG4108QVM	0.000348453	0.00046618
P Inorganic ion transport and metabolism; ENOG4105DH3	0.000347117	0.000180151
S Function unknown; ENOG4105V0F	0.000328789	0.000247238
S Function unknown; ENOG4108S9K	0.000307536	0.00033249

**Table 4:** The relationship between differential species and traditional biomarkers in GD patients.

	uc_*Bacteroide*		*Thetaiotaomicron*		*Dorei*		*Fragilis*		*Cellulosilyticus*	
	*r*	*p*-Value	*r*	*p*-Value	*r*	*p*-Value	*r*	*p*-Value	*r*	*p*-Value
Total bile acid	0.062	0.735	0.299	0.096	−0.041	0.822	0.208	0.253	0.169	0.356
Alkaline phosphatase	0.005	0.771	0.245	0.149	0.042	0.806	0.126	0.464	−0.080	0.645
γ-Glutamyl transpeptidase	−0.232	0.174	0.021	0.904	−0.067	0.700	0.040	0.819	−0.225	0.188
Direct bilirubin	−0.197	0.250	−0.021	0.903	−0.304	0.071	−0.113	0.513	−0.325	0.053
Total bilirubin	−0.256	0.131	−0.105	0.543	−0.395	0.017	−0.200	0.243	−0.416	0.012
Alpha-l-fucosidase	0.154	0.493	−0.098	0.665	0.296	0.181	−0.186	0.406	−0.197	0.379
Urea nitrogen	0.125	0.475	0.021	0.906	0.104	0.551	−0.156	0.370	0.086	0.624
Creatinine	0.151	0.386	0.282	0.101	0.180	0.301	−0.075	0.671	−0.044	0.803
Uric acid	0.030	0.863	0.248	0.151	0.047	0.788	−0.256	0.138	−0.116	0.506
Cystatin C	−0.264	0.158	−0.016	0.935	−0.307	0.099	−0.402	0.027	−0.065	0.734
Glutamate dehydrogenase	0.115	0.601	0.399	0.060	0.158	0.472	0.058	0.791	−0.001	0.995
Fibronectin	−0.290	0.203	−0.269	0.239	0.040	0.862	−0.113	0.626	0.047	0.841
Cholyglycine	0.015	0.937	0.238	0.198	−0.042	0.823	0.222	0.230	0.047	0.802
Total protein/albumin	0.301	0.066	0.162	0.331	0.102	0.544	0.150	0.368	0.014	0.934
Total protein	−0.024	0.889	−0.004	0.983	0.133	0.440	0.182	0.288	−0.089	0.606
Albumin	0.210	0.218	0.204	0.233	0.282	0.096	0.248	0.145	0.041	0.810
Globulin	−0.137	0.440	0.006	0.974	−0.056	0.755	0.053	0.767	−0.098	0.582
Prealbumin	0.077	0.741	0.483	0.027	0.288	0.205	0.336	0.136	−0.022	0.924
Alanine aminotransferase	0.024	0.887	0.096	0.572	0.113	0.507	−0.124	0.466	0.108	0.524
Aspartate aminotransferase	0.003	0.988	−0.068	0.688	−0.039	0.821	0.025	0.884	0.040	0.812
Lactate dehydrogenase	−0.108	0.642	−0.006	0.978	0.009	0.969	0.083	0.722	−0.029	0.900

## Data Availability

All raw sequences were deposited in the NCBI Sequence Read Archive under accession number PRJNA999028.
